# Supplementary material for: Cloud BioLinux: pre-configured and on-demand bioinformatics computing for the genomics community
Source: BMC Bioinformatics. 2012 Mar 19;13:42. doi: 10.1186/1471-2105-13-42 (PMC3372431; doi:10.1186/1471-2105-13-42)
Supplement: Additional file 1 — Supplementary 1 Cloud BioLinux software documentation in the form of a mini, self-contained website. Users need to download and uncompress the .zip file, and open through a web browser the "index.html" file available on the main directory. (ZIP 1823 kb). [file 1471-2105-13-42-S1.ZIP › Cloud-BioLinux-Package-Documentation/docs/dba.html]

Bio-Linux Software Documentation Pages

Back to search form

## dba

|  |  |
| --- | --- |
| Name | dba |
| Description | **dba** - standing for Dna Block Aligner, was developed by Niclas Jareborg, Richard Durbin and Ewan Birney for characterising shared regulatory regions of genomic DNA, either in upstream regions or introns of genes.  The idea was that in these regions there would a series of shared motifs, perhaps with one or two insertions or deletions but between motifs there would be any length of sequence (part of the Wise2 package).  Wise2 is a package focused o­n comparisons of bio polymers, commonly DNA sequence and protein sequence. Wise2's particular forte is the comparison of DNA sequence at the level of its protein translation. This comparison allows the simultaneous prediction of say gene structure with homology based alignment.  The main programs within Wise2 are:-  **genewise** compares a protein sequence or a protein profile HMM to a dna sequence.  **genewisedb** is the database searching version of genewise. It takes a database of proteins and compares it to a database of dna sequences (genewisedb - search modes).  **estwise** runs very much like genewise with basically a subset of options. For completeness they are all listed below (estwise - options: dna/protein).  **estwisedb** is the database searching version of the estwise program. Like estwise, it has the same sort of running modes as genewisedb, but with more limited options.  These programmes all have basically the same running mode:-  `%genewise protein-file dna-file`  Other programs:-  **dba** - standing for Dna Block Aligner, was developed by Niclas Jareborg, Richard Durbin and Ewan Birney for characterising shared regulatory regions of genomic DNA, either in upstream regions or introns of genes.  **psw** - a short and sweet program for calculating smith waterman alignments quickly. It was mainly written as C driver to test the underlying code which is more useful in things like the Perl port.  **pswdb** - protein smith waterman database searching was written by Richard Copley using the underlying Wise2 libraries psw - options.  A number of options are common to these programs from the point of view of how they run:   - - help    verbose help of all options. - - version    show version and compile info. - - silent    No messages o­n stderr, whether reports or warnings. - - quiet    No reports or information messages o­n stderr. - - erroroffstd    No warning messages to stderr, but reports are still issued. - - errorlog    [file] Log warning messages to file.  An example file can be found o­n IVGFS in  `/usr/software/sampledata`  A manual can be found at http://www.ebi.ac.uk/Wise2/  **References:**  Birney E, Durbin R. Using GeneWise in the Drosophila annotation experiment Genome Res. 2000 Apr;10(4):547-8. [Entrez] |
| Homepage | http://www.ebi.ac.uk/Wise2/index.html |
| Remote Documentation | http://www.ebi.ac.uk/Wise2/doc\_wise2.html |

|  |
| --- |
| Please see Remote Documentation |
